# The Impacts of Dietary Change on Greenhouse Gas Emissions, Land Use, Water Use, and Health: A Systematic Review

**DOI:** 10.1371/journal.pone.0165797

**Published:** 2016-11-03

**Authors:** Lukasz Aleksandrowicz, Rosemary Green, Edward J. M. Joy, Pete Smith, Andy Haines

**Affiliations:** 1 Dept. of Population Health, London School of Hygiene & Tropical Medicine, London, United Kingdom; 2 Leverhulme Centre for Integrative Research on Agriculture & Health, London, United Kingdom; 3 Institute of Biological and Environmental Sciences, University of Aberdeen, Aberdeen, United Kingdom; 4 Dept. of Social & Environmental Health Research, London School of Hygiene & Tropical Medicine, London, United Kingdom; Indiana University Bloomington, UNITED STATES

## Abstract

Food production is a major driver of greenhouse gas (GHG) emissions, water and land use, and dietary risk factors are contributors to non-communicable diseases. Shifts in dietary patterns can therefore potentially provide benefits for both the environment and health. However, there is uncertainty about the magnitude of these impacts, and the dietary changes necessary to achieve them. We systematically review the evidence on changes in GHG emissions, land use, and water use, from shifting current dietary intakes to environmentally sustainable dietary patterns. We find 14 common sustainable dietary patterns across reviewed studies, with reductions as high as 70–80% of GHG emissions and land use, and 50% of water use (with medians of about 20–30% for these indicators across all studies) possible by adopting sustainable dietary patterns. Reductions in environmental footprints were generally proportional to the magnitude of animal-based food restriction. Dietary shifts also yielded modest benefits in all-cause mortality risk. Our review reveals that environmental and health benefits are possible by shifting current Western diets to a variety of more sustainable dietary patterns.

## Introduction

There is an urgent need to curb the degradation of natural resources and to limit global warming to less than 2°C, while providing a nutritious diet to a growing and changing world population [1, 2]. Agriculture is responsible for up to 30% of anthropogenic greenhouse gas (GHG) emissions, about 70% of freshwater use, and occupies more than one-third of all potentially cultivatable land [2, 3], with animal-based foods being particularly major contributors to these environmental changes [4]. These impacts present challenges for improving global health and development, by exacerbating climate change, driving biodiversity loss and soil degradation, and increasing freshwater scarcity [2, 5]. At the same time, dietary risk factors are major contributors to the burden of non-communicable diseases through inadequate intake of fruit, vegetables, nuts and seeds, and dietary fibre, together with high consumption of red and processed meat [6].

The Rockefeller Foundation-Lancet Commission on Planetary Health suggested that there is major potential for dietary changes to improve health and reduce the environmental impacts of food production [2]. The United Nations Food and Agriculture Organization (FAO) defines sustainable diets as those which are healthy, have a low environmental impact, are affordable, and culturally acceptable [7]. A growing body of research has analysed the environmental impacts in high-income countries (HICs) of adopting diets that are proposed to lower the environmental footprint of food production, often referring to these as sustainable diets [8–11]. A variety of sustainable dietary patterns have been suggested, including vegetarian and Mediterranean, as well as following national dietary recommendations. Such diets may deliver health and environmental benefits due to partial replacement of animal products with plant-based foods [8, 12], and thus, adopting sustainable diets may play an important role in achieving a number of the Sustainable Development Goals (SDGs).

However, widespread policy action is lacking on integrating environmental and nutritional priorities [13]. This may be limited by the lack of collated data and clear summaries of the environmental and health impacts of shifts to sustainable diets—with the body of research using a variety of proposed sustainable diets, and most studies focusing on only one aspect of sustainability—and therefore uncertainty about the possible magnitude of impacts.

We systematically review the evidence of the impacts of adopting sustainable diets on GHG emissions, agricultural land requirement, and water use, and compare the environmental and health effects between various types of sustainable dietary patterns. Our analysis aims to substantially expand on two previous reviews [14, 15], as a large number of studies in this area have been published since then, and we also include grey literature, and the additional indicators of water use and health impacts.

## Methods

### Search strategy and selection criteria

**Fig 1:**
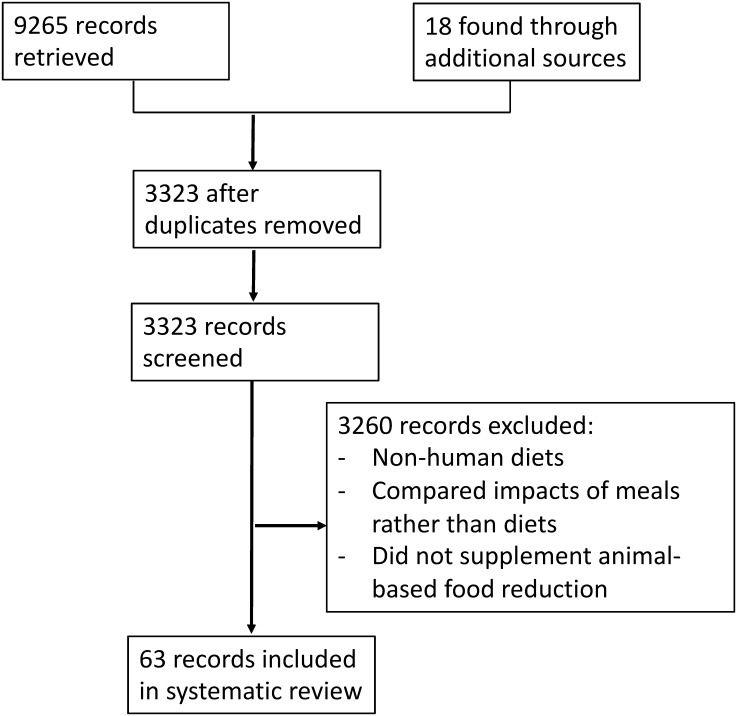
Selection of eligible studies.

We conducted a systematic review of studies measuring the environmental impacts of shifting current average dietary intake to a variety of proposed sustainable dietary patterns, and our review is current as of 10^th^ June 2016. We followed PRISMA quality guidelines [16]. The environmental impacts we considered were GHG emissions, land use and water use. Scopus, ProQuest, PubMed, Web of Science, and Science Direct databases were searched for articles. Peer-reviewed studies with English-language abstracts from any region were eligible, as well as grey literature such as conference abstracts and reports. Studies were screened for inclusion independently by two reviewers (LA, EJ), and were reviewed for other relevant references (Fig 1).

Inclusion criteria for studies were as follows: quantifying changes in GHG emissions, land use, or water use, between average population-level dietary intake and proposed sustainable dietary patterns; using dietary or consumer expenditure surveys, or food balance sheets to inform the baseline diets; and, using baseline dietary data from 1995 onwards. The three environmental indicators were selected based on an initial screening of available indicators in the literature. Studies were excluded if they evaluated the impacts of single food items or meals rather than dietary patterns, or used alternative diets targeting meat or dairy reduction without compensating for this decrease in energy intake with intake of other foods. Our literature search identified a related theme of research on carbon taxes, which have been proposed as a tool to reduce GHG emissions through influencing consumer food choice and therefore dietary patterns. We did not include these studies in our main analysis as the resulting diets did not fully align with the common dietary patterns found across all other retrieved studies. However, the discussion section summarises findings from the studies that investigated the effect of carbon taxes on dietary GHG emissions.

The following parameters were extracted from studies: country or region, year of baseline diet, methods and sources of environmental impact data, type of sustainable diet(s) measured, environmental impacts of baseline and sustainable diets, if GHG emissions included those from land use change, health impacts, degree of change for the sustainable diet (e.g., amount of meat reduction), whether sustainable dietary patterns were self-selected within studies (dietary patterns as eaten by study participants, as opposed to modelled or designed by study authors), and energy content of baseline and sustainable diets.

### Analysis and quality assessment

Average population-level intakes in the reviewed studies were taken as the baseline diet, with each comparison between a baseline diet and a given sustainable diet categorised as an individual scenario. In each scenario, differences in environmental impacts between baseline and sustainable diets were quantified as the relative differences in carbon dioxide-equivalent GHG emissions (kg CO_2_eq/capita/year, which is an adjusted indicator including CO_2_, N_2_O, and CH_4_), land use (m^2^/capita/year), and water use (L/capita/day). Where studies reported impacts in absolute amounts, we converted these to relative differences. Impacts were stratified by sustainable dietary pattern type, and by environmental indicator. Environmental impact data using life cycle analysis (LCA) often do not include measures of variance, and therefore the reviewed studies did not provide confidence intervals for environmental impacts. Impacts did also not include systemic environmental feedbacks. Differences in environmental impacts between diet types were assessed using medians, and visualised using box and whisker blots. We converted any health effects originally reported in absolute terms to relative changes, by using appropriate population totals from the Global Burden of Disease Study [17]. We used a sign test to check if the number of instances where the direction of impact changed after adopting sustainable diets was statistically significantly different than what would be expected due to chance alone.

Study quality was assessed through three requirements: modelling the baseline diet on dietary intake surveys rather than food availability or expenditure; a description of the source and methods of the environmental impact data used; and that differences in the energy content of baseline and sustainable diets were within 5%. This latter cut-off was used as as some studies aimed for an isocaloric design between compared diets, but due to modeling logistics, some minor caloric differences remained. These quality measures were selected since food balance sheets or expenditure-based surveys may differentially under- or over-estimate consumption of certain food groups [18], while the effect of not standardising calories may attribute environmental impacts to a reduction in absolute food intake rather than choice of food type. The potential for bias in the results was assessed by removing those studies that did not meet the above requirements, and using Spearman coefficients to compare the ranking of sustainable diet types before and after removal of studies, as well as a sign test for the direction of impact.

The review protocol, with additional information and specific search terms, is available in S1 File. Analyses were performed, and graphs made, using STATA version 14.

## Results

A total of 210 scenarios were extracted from 63 studies. Of these, 204 scenarios were modelled on national-level diets in HICs, one on a city in a middle-income country, and five on global dietary patterns (S1a–S1c Table) [8–11, 19–77]. Fourteen studies came from grey literature. Fourteen sustainable dietary patterns were proposed: vegetarian, vegan, pescatarian, replacing ruminant with monogastric meat, balanced energy intake, following healthy guidelines, Mediterranean diet, New Nordic diet, and meat reduction, with other sub-scenarios such as type of food supplemented by meat reduction, and healthy guidelines with further optimisation (Table 1). Several studies designed sustainable diets by starting with national healthy guidelines and optimised the balance of foods further, through linear programming [9, 11, 53, 56, 63, 66, 72, 75] or manually [32, 34, 38, 45, 54, 55, 59, 67], to generate additional environmental benefits; these scenarios have been termed “healthy guidelines plus further optimisation”. Balanced energy intake were scenarios where the average current diet was scaled down to recommended caloric intakes without changing the mix of food groups eaten. The category of meat replacement with mixed foods indicates dairy and plant-based food.

**Table 1:** Description of the number of reviewed scenarios, by type of sustainable dietary pattern and environmental indicator.

Sustainable diet type	Environmental impact		
	GHG emissions	Land use	Water use
Vegan	14	6	1
Vegetarian	20	7	9
Ruminants replaced by monogastric meat	6	3	1
Ruminants replaced by monogastric + no dairy	1	-	-
Meat partially replaced by plant-based food	8	4	-
Meat partially replaced by dairy products	3	1	-
Meat partially replaced by mixed food	7	1	-
Meat + dairy partially replaced by plant-based food	5	3	3
Balanced energy intake	6	2	1
Healthy guidelines	21	10	9
Healthy guidelines + further optimisation	16	5	4
Mediterranean	8	5	4
New Nordic Diet	3	1	-
Pescatarian	6	4	2
**Total**	124	52	34

Of the 210 scenarios, 197 showed a reduction in environmental impacts when switching from baseline to alternative dietary patterns (sign test: p<0·0001), while thirteen scenarios showed an increase or no impact. The median changes in GHG emissions, land use, and water use, across all sustainable diet types, were -22%, -28%, and -18%, respectively. The largest environmental benefits across indicators were seen in those diets which most reduced the amount of animal-based foods, such as vegan (first place in terms of benefits for two environmental indicators), vegetarian (first place for one indicator), and pescatarian (second and third place for two indicators).

**Fig 2:**
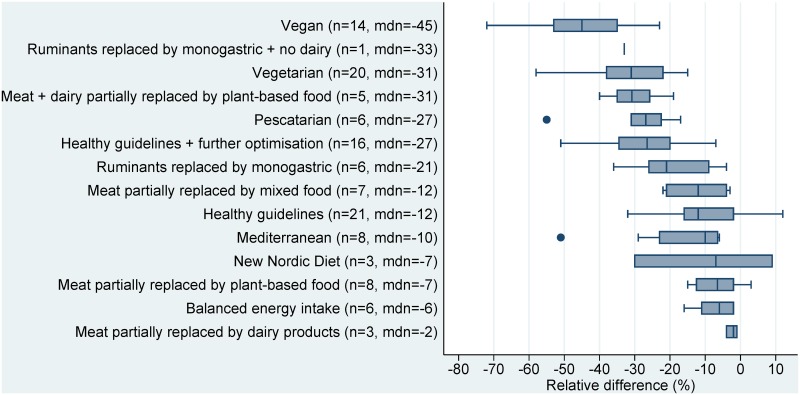
Relative differences in GHG emissions (kg CO_2_eq/capita/year) between current average diets and sustainable dietary patterns. Note: n = number of studies, mdn = median.

**Fig 3:**
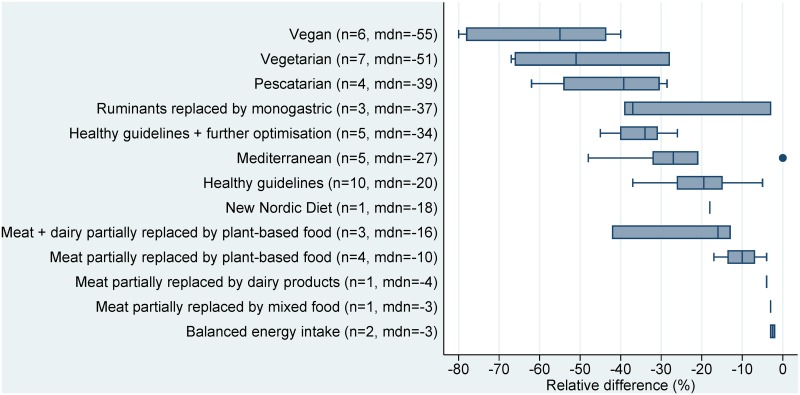
Relative differences in land use (m^2^/capita/year) between current average diets and sustainable dietary patterns. Note: n = number of studies, mdn = median.

**Fig 4:**
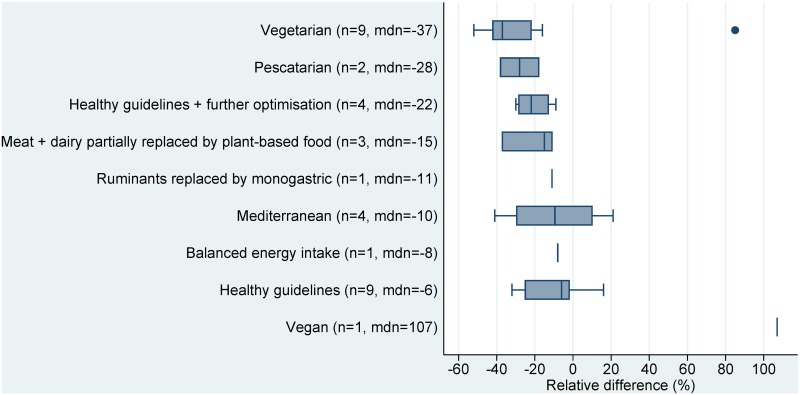
Relative differences in water use (L/capita/day) between current average diets and sustainable dietary patterns. Note: n = number of studies, mdn = median. The lower and upper bounds of the boxes represent the 1^st^ and 3^rd^ quartiles, respectively, and the line within is the median. Whiskers show the minimum and maximum range, excluding outliers, which are shown as dots, and represent values more than 1.5 times the 1^st^ and 3^rd^ quartiles.

The ranking of sustainable diet types showed similar trends for land use and GHG emissions, with vegan diets having the greatest median reductions for both indicators (-45% and -51%, respectively), and scenarios of balanced energy intake or meat partly replaced with dairy, having the least benefit. Although the water use scenarios had smaller sample sizes, they showed somewhat similar trends across sustainable diet types, with vegetarian diets having the largest benefit (median -37%), though with the notable exception of the single vegan scenario showing an increase in water use (+107%) (Figs 2–4).

We assessed the sensitivity of our findings to study quality. Excluding papers that did not meet the three quality criteria resulted in minor differences in findings. The overall direction of impact did not change (sign test: p = 0·5), and the ranking of sustainable diet types had strong correlation with the full list of studies for GHG emissions and land use (Spearman’s rho: 0·93, p<0·0001; 0·83, p = 0·003, respectively). The correlation between rankings was not significant for water use (Spearman’s rho: 0·20, p = 0·8); this was likely due to the number of scenarios decreasing from 34 to 4 when removing lower-quality studies (S2 Table). The magnitude of environmental impacts for diets stayed similar (S1a–S1c Fig). Excluding grey literature sources had little effect, with the overall ranking of sustainable dietary patterns showing almost no change across the environmental indicators (sign test: p = 0.21; Spearman’s rho: 0·96–1·0, p<0·0001), (S2 Table, S2a–S2c Fig).

Analyses of the health effects of sustainable diets were limited. Within the seven studies reporting health effects of adopting sustainable diets, 11 out of the 14 sustainable diet types were modelled, with a single estimate of all-cause health impacts for all but two of the 11 diet types. Most studies assessed the reduction in mortality risk from adopting a sustainable diet, either by all-cause or cause-specific mortality (Table 2). All studies showed positive health effects, ranging from <1% reduction in estimated mortality risk for vegetarian diets, to 19% for vegan diets, though some of these were not statistically significant. The magnitude of health effects across the sustainable dietary patterns did not show a statistical association with that of environmental benefit.

**Table 2:** Health effects of sustainable dietary patterns.

Study		Country	Sustainable diet type	Health indicator	Change in health indicator (95%CI)*
Sabate 2015	^74^	US/Canada	Vegan	All-cause mortality rate	19.2%
Soret 2014	^50^	US/Canada	Vegetarian	All-cause mortality risk	9% (0–17)
Tilman 2014	^8^	Globally	Vegetarian	All-cause mortality risk	<1% (0–2)**
Sabate 2015	^74^	US	Vegetarian	All-cause mortality rate	15.9%
Aston 2012	^21^	UK	Meat partially replaced by mixed food	CHD risk (men)	9.7% (-3.5–22)
Aston 2012	^21^	UK	Meat partially replaced by mixed food	CHD risk (women)	6.4% (-1.8–14.3)
Aston 2012	^21^	UK	Meat partially replaced by mixed food	Diabetes mellitus risk (men)	12% (-4.5–22.7)
Aston 2012	^21^	UK	Meat partially replaced by mixed food	Diabetes mellitus risk (women)	7.5% (0.5–14.5)
Aston 2012	^21^	UK	Meat partially replaced by mixed food	Colorectal cancer risk (men)	12.2% (6.4–18.0)
Aston 2012	^21^	UK	Meat partially replaced by mixed food	Colorectal cancer risk (women)	7.7% (4.0–11.3)
Soret 2014	_50_	US/Canada	Meat partially replaced by mixed food	All-cause mortality risk	14% (4–23)
Sabate 2015	_74_	US/Canada	Meat partially replaced by mixed food	All-cause mortality rate	7.2%
Biesbroek 2014	^25^	Netherlands	Meat partially replaced by plant-based food	All-cause mortality risk	10% (3–16)
Biesbroek 2014	^25^	Netherlands	Meat partially replaced by dairy	All-cause mortality risk	6% (-4-14)
Tilman 2014	^8^	Globally	Mediterranean	All-cause mortality risk	18% (17–19)
Sabate 2015	^74^	US/Canada	Pescatarian	All-cause mortality rate	17.6%
Milner 2015	^79^	UK	Healthy guidelines	Years of life lost^+^	6%
Milner 2015	^79^	UK	Healthy guidelines + further optimisation	Years of life lost^+^	7%
Scarborough 2012	_80_	UK	Meat, dairy partially replaced by plant-based food	Deaths averted	6%
Scarborough 2012	^80^	UK	Ruminants replaced by monogastric	Deaths averted	<1%

*Percentages refer to reductions in health indicators, except for deaths averted

**Mortality risk reduction by cause: cancer 10%, coronary heart disease 20%, type 2 diabetes 42%

^+^Years of life lost, at year 30 (after adoption of the sustainable diet scenario)

## Discussion

Our review showed that reductions above 70% of GHG emissions and land use, and 50% of water use, could be achieved by shifting typical Western diets to more environmentally sustainable dietary patterns. Medians of these impacts across all studies suggest possible reductions of between 20–30%. This review is the most recent and comprehensive to date, and the first to compare impacts across GHG emissions, land use, and water use. This work supports the conclusions of previous reviews in this area[14, 15] which also pointed to the potential for reductions in GHG emissions and land use from dietary change. However, our review substantially expands the number of studies and dietary patterns assessed, and includes grey literature. Our use of multiple environmental indicators also highlights possible trade-offs across the proposed dietary patterns, both in magnitude and direction of the environmental impacts.

Underlying environmental data in the studies (where shown) on the land use, GHG emissions, and water use impacts from the production of food items showed decreasing impacts, from greatest to least, across ruminant meat, other meat, dairy, and plant-based foods [9, 23, 24, 32, 39, 46, 51, 60, 78]. Therefore, the large majority of scenarios showed decreased environmental footprints from replacement of plant- with animal-based foods. However, we note some exceptions. Eleven scenarios out of 210 showed higher environmental impacts of shifts to sustainable diets [32, 38, 55, 60, 62, 63, 73], with two scenarios having no effect [60, 63]. In some studies, the underlying data on environmental footprints for plant-based foods were similar to or higher than for some meats (e.g. water use per calorie of nuts, fruits and vegetables being higher than several animal-based foods [38, 62]). Therefore, replacing calories from meat reduction scenarios with increased plant-based foods produced higher water footprints or GHG emissions in some cases [38, 55, 60, 62, 73]. A more thorough review of GHG impacts across food items by Tilman and Clark confirms these overall trends and possible exceptions [8], though comparisons of impacts between any specific food items are likely to vary by region and food production context. The make-up of the alternative dietary patterns was also a factor in instances of higher environmental impacts. For example, in studies assessing shifts to US dietary guidelines [33, 62], increases in footprints appeared to be driven in part by the particular US recommendations to greatly increase dairy intake. In Vieux et al., meat reduction supplemented isocalorically by fruit and vegetables showed an increase in emissions, while a secondary scenario (and arguably more realistic) of replacement with mixed foods (grains, vegetables, and dairy) saw a net decrease [60]. Such scenarios highlight some of the complexity involved in assessing environmental sustainability of diets, and the context- and region-specific nature of such assessments.

Studies modelling the health impacts of shifts from typical Western diets to sustainable dietary patterns showed modest health gains from reductions in mortality rates and risks [8, 21, 25, 50, 79, 80]. There was no statistical association between the magnitude of environmental and health benefits, though the number of studies modelling health scenarios was limited. A recent review of health impacts of low-carbon diets confirms our findings [81]. The health benefits of sustainable diets may derive from increases in fruit and vegetable consumption and reductions in red and processed meat [6], as well as lower overall calorie intake for those individuals at risk of over-nutrition. However, health and environmental priorities may not always converge, for example, as sugar may have low environmental impacts per calorie relative to other foods, and some fruit or vegetables may have higher GHG emissions per calorie than dairy and non-ruminant meats [39, 46, 60, 78]. Intake of fish, the consumption of which is still below recommended levels in many regions, will also have to be reconciled with the fragility of many global wild-catch fisheries and unsustainable practices in aquaculture [82].

This review had several limitations. The available studies were from a narrow range of HICs with different baseline dietary patterns, and used largely HIC-specific environmental data sources. The results may therefore only be generalizable to HICs. The data on environmental impacts did not provide measures of variance, and we were limited to graphical and non-parametric statistical methods to assess the differences between sustainable dietary patterns. We were also unable to rule out any effects of publication bias in the literature. The use of environmental indicators varied across studies, such as whether blue, green or grey water (or a combination) was used, and whether GHG emissions included the often significant emissions from land use change. Our use of relative differences in the analysis helped to accommodate some of the differences in methodology across studies, and despite this heterogeneity, our resulting median impacts produced internally consistent and plausible trends; for example, vegan diets having greater reductions in GHG emissions than vegetarian; greater benefits from reducing meat and dairy consumption compared to meat alone; and replacing meat with dairy having little benefit.

There is an increasing body of evidence on which to base the integration of environmental priorities into dietary recommendations. Several of these dietary patterns are already promoted through public health efforts, such as the healthy dietary guidelines, the Mediterranean diet [83, 84], and the New Nordic Diet [85]. Brazil and Sweden have also recently made efforts to add environmental priorities into dietary guidelines [86, 87]. Additionally, our literature search retrieved studies measuring environmental impacts of potential dietary shifts resulting from carbon taxes on food products [88–91]. These studies calculated reductions in GHG emissions on average of about 6–9%, supporting our conclusions that dietary change can reduce environmental impacts, and offering a policy route for achieving these aims.

Several considerations regarding environmentally sustainable eating are worth noting. Firstly, the production of food (i.e. the growing of crops and raising of livestock) is the primary driver of environmental impacts, as opposed to later stages such as transport and processing [92, 93]. While local and seasonal diets have advantages such as protecting local economies and crop diversity, efforts to reduce dietary-related environmental impacts should focus on reducing animal-based foods in high-consuming societies.

However, complete removal of animal-source foods is not realistic in many cultures and may have important health implications. Meat and dairy are high-quality sources of protein and micronutrients, and ensuring adequate bioavailable supply of these is essential for public health [94]. This review has largely focused on population-level intake, and further work should consider dietary requirements of sub-population groups, including children and women of child-bearing age. Moderate consumption of pork and poultry may be consistent with a more sustainable diet, as these have lower environmental impacts than ruminant meat. Additionally, raising of livestock in some regions allows humans to derive nutritional benefit from non-arable land, or to utilize crop residues and food waste [95].

Lastly, shifts to sustainable diets must be affordable and desirable for consumers. Studies have shown that large reductions in GHG emissions are possible without complete exclusion of animal products [9], and studies using self-selected sustainable diets imply these could be culturally appropriate for at least some individuals [24, 27, 49, 50, 96]. However, extending these patterns to the majority of the population will require large efforts. In HICs, healthy foods are often more expensive than unhealthy ones [97], and rebalancing these relative prices will be critical to help steer consumers towards more sustainable choices [98].

Our estimates would benefit greatly from more comprehensive data, and further work should generate regional and food-specific environmental impacts, including for fisheries and aquaculture, as well as measures of variance. A limited number of studies calculated a reduction in nitrogen and phosphorus water contamination from sustainable eating patterns [10, 52], and further studies on these and other indicators are required. The resilience of sustainable diets to future environmental changes, such as rainfall patterns and the effect of rising carbon dioxide on nutritional quality of food, needs to be assessed [99]. Little is also known about the environmental impacts of different dietary patterns in low- and middle-income countries. The reviewed diets cannot be designated sustainable in an absolute sense, as this will depend on population growth, evidence about planetary boundaries, and assumptions about other environmental trends [2], and more work is necessary to define sustainable diets along a more comprehensive range of environmental, economic and social indicators.

The impacts of sustainable diets are linked to a number of SDGs, including goals on sustainable agricultural practices, health, water use, and climate change. Promotion and uptake of these diets could therefore offer a route, along with other strategies, to achieving several of the SDGs.

Across a large and heterogeneous set of studies, several policy implications are clear: environmental benefits are possible in HICs from shifting current diets to a variety of more sustainable dietary patterns; environmental benefits are largely proportional to the magnitude of meat (particularly from ruminants) and dairy reduction; and a redoubling of efforts to promote the uptake of diets that support these changes could bring environmental and health benefits.

## Supporting Information

S1 Fig A-C. Relative difference in A) GHG emissions (kg CO_2_eq/capita/year), B) land use (m^2^/capita/year), and C) water use (L/capita/day), between current average diets and sustainable dietary patterns, after excluding studies that did not meet quality criteria.
(DOCX)

S2 Fig A-C. Relative difference in A) GHG emissions (kg CO_2_eq/capita/year), B) land use (m^2^/capita/year), and C) water use (L/capita/day), between current average diets and sustainable dietary patterns, after excluding grey literature.
(DOCX)

S1 File Systematic review protocol. (DOCX)

S1 Table A-C. Included studies, study details, and environmental impacts for GHG emissions, land use, and water use.
(XLSX)

S2 Table Number of sustainable diet types showing greater, lower, or neutral environmental impacts, and Spearman’s coefficients, after removal of grey literature and studies that did not meet quality criteria. (XLSX)
