# Supplementary material for: The Impacts of Dietary Change on Greenhouse Gas Emissions, Land Use, Water Use, and Health: A Systematic Review
Source: PLoS One. 2016 Nov 3;11(11):e0165797. doi: 10.1371/journal.pone.0165797 (PMC5094759; doi:10.1371/journal.pone.0165797)
Supplement: S2 Fig — A-C. Relative difference in A) GHG emissions (kg CO2eq/capita/year), B) land use (m2/capita/year), and C) water use (L/capita/day), between current average diets and sustainable dietary patterns, after excluding grey literature. (DOCX) [file pone.0165797.s002.docx]

**S2 Fig. A. Relative difference in GHG emissions (kg CO_2_eq/capita/year) between current average diets and sustainable dietary patterns, after excluding grey literature***

**S2 Fig. B. Relative difference in land use (m^2^/capita/year) between current average diets and sustainable dietary patterns, after excluding grey literature***

**S2 Fig. C. Relative difference in water use (L/capita/day) between current average diets and sustainable dietary patterns, after excluding grey literature***


*n=number of studies; mdn=median
