# Supplementary material for: The Impacts of Dietary Change on Greenhouse Gas Emissions, Land Use, Water Use, and Health: A Systematic Review
Source: PLoS One. 2016 Nov 3;11(11):e0165797. doi: 10.1371/journal.pone.0165797 (PMC5094759; doi:10.1371/journal.pone.0165797)
Supplement: S1 File — (DOCX) [file pone.0165797.s003.docx]

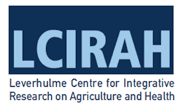

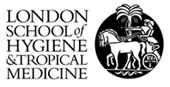


Systematic Literature Review Protocol

Environmental Impacts of Sustainable Diets

Leverhulme Centre for Integrative Research on Agriculture and Health (LCIRAH) / London School of Hygiene and Tropical Medicine (LSHTM)

1^st^ June 2015

Review team:

Lukasz Aleksandrowicz (lead) – LCIRAH / LSHTM

Dr Edward Joy – LCIRAH / LSHTM

Dr Rosemary Green – LCIRAH / LSHTM

Professor Andy Haines - LSHTM

**Environmental impacts of sustainable diets: a systematic review**

**1) Research question**

To review the diet-related environmental impacts of shifting food consumption from average current diets to sustainable alternative diets, for available countries.

**2) Background**

Agricultural systems globally exert large environmental impacts. Agriculture contributes to about 20% of global greenhouse gas emissions [1], 70% of water use, and occupies more than a third of all land [1,2]. Shifts to more sustainable diets may therefore have the potential to mitigate some of these impacts.

Many studies have calculated the environmental impacts of sustainable diets. However, there are still varying opinions on what a sustainble diet may specifically look like, as well as some criticism of the effectiveness of sustainable eating [3,4]. This has likely detracted from meaningful policy change, an example of which has been the recent opposition to the US Dietary Guidelines Advisory Committee’s recommendation of including environmental considerations in dietary guidelines [5].

Several previous reviews have been conducted on sustainable diets, however, these have largely been general literature reviews commenting on the models or metrics [7,8], or have not incorporated all available evidence on the estimates of environmental impacts [6]. The current proposed analysis will be a systematic review to update all available evidence on environmental impacts of dietary shifts.

**3) Aims**

This systematic review will primarily answer two questions:

- what are the various definitions of sustainable diets proposed in the literature?
- what are the environmental impacts of these sustainable diets, measured in terms of greenhouse gas emissions, land use, and water use, compared to current diets?

**4) Search strategy**

The following search concepts will be used:

1. (meat OR "sustainable diet*" OR "diet* pattern*" OR "diet* choice*" OR “diet* change*” OR “diet* recommendation*” OR “diet* guideline*” OR “diet* scenario*” OR “ diet* type*” OR “Mediterranean diet*” OR "food choice*" OR "food consumption" OR "health* diet*" OR “health* eat*”) AND (greenhouse OR GHG OR ecological OR “environment* sustain*” OR "global warming" OR climate OR water OR land OR “land-use” OR "environment* impact*" OR footprint*)
2. (diet*) AND (greenhouse OR GHG OR ecological OR “environment* sustain*” OR "global warming" OR climate OR water OR land OR “land-use” OR "environment* impact*" OR footprint*)
3. ("sustainable diet*" OR "diet* pattern*" OR "diet* choice*" OR “diet* change*” OR “diet* recommendation*” OR “diet* guideline*” OR “diet* scenario*” OR “ diet* type*” OR “Mediterranean diet*” OR "food choice*" OR "food consumption" OR "health* diet*" OR “health* eat*”) AND (environment*)
4. AND NOT (biogas* OR bioenergy OR manure OR antioxidant* OR antibiotic* OR immune* OR cropping OR yield OR "E. coli" OR safety OR metabolic OR management OR *parasite* OR *forestry OR “crop-livestock” OR mice OR broiler*)

The databases to be searched will include Scopus, PubMed, Web of Science, Science Direct, ProQuest, and Google Scholar. Peer-reviewed studies will be included, as well as appropriate grey literature such as dissertations, conference proceedings, and reports, which meet the inclusion criteria described below. Studies will be hand-searched for other relevant references.

***Inclusion criteria***

- Quantifying environmental indicators in the form of greenhouse gas emissions, land use, or water use, between average population-level dietary intake and alternative diets
- Using dietary surveys or food balance sheets to inform the population-level baseline diets
- Studies conducted between 2000-2015, and using consumption or intake data from 1995 onwards
- English language

***Exclusion criteria***

- Comparison of environmental impacts of food items or single meals rather than diets
- Any alternative diets targeting meat or dairy reduction which did not supplement this decrease with other foods
- Review articles will be excluded, as well as multiple publications of the same study (e.g., results being published in a journal as well as report). In any such cases, the peer-reviewed source will be used.

**5) Study quality and risk of bias**

Study quality will be assessed through a checklist [9] that includes the following components:

- Current, average diets for comparison are based on dietary intake surveys at a national or sub-national level rather than national food balance sheets
- Description and source of the environmental impact data used in the model
- Baseline and comparison sustainable diets are isocaloric

A sensitivity analysis will be performed with the exclusion of any studies not meeting the above components.

**6) Data extraction**

Titles and abstracts will be used to filter papers into potential and non-relevant groups. Full-text versions will be accessed for potential papers. Potential papers meeting all inclusion criteria above will be extracted for details on the following variables: study location, years, source of environmental impacts data, type of sustainable diet measured, environmental impacts, degree of change, if any, for the sustainable diet (e.g., amount of meat reduction), and energy content of baseline and alternative diets. Data will be stored in excel. Half of the records will be screened and have data extracted independently by a second reviewer, to limit exclusion of relevant studies. Half of relevant studies will have also have data extraction checked to limit any errors.

**7) Data analysis**

Differences in environmental impacts between baseline and alternative diets will be compared, stratified by type of alternative diet. As standard errors are not available for the types of studies being reviewed, statistical tests will not be performed. Results will be displayed with box plots, using means and ranges to compare effects across types of sustainable diet.

**8) Conclusions**

This review will quantitatively assess the environmental impacts between current and alternative sustainable diets, for available countries. The results will provide a synthesis of possible iterations of sustainable diets, and their estimated environmental impacts, to help inform policy decisions around integrating environmental considerations into dietary guidelines.

References

[1] IPCC. Working Group 3 - Mitigation of Climate Change: Chapter 11: Agriculture, Forestry and Other Land Use (AFOLU). Cambridge, United Kingdom, and New York City, USA, 2014.

[2] Food and Agriculture Organization of the United Nations (2010) Water withdrawal by sector. AQUASTAT database. <http://www.fao.org/nr/aquastat>

[3] Auestad N, Fulgoni VL. What current literature tells us about sustainable diets: emerging research linking dietary patterns, environmental sustainability, and economics. Advances in Nutrition 2015(6):19**–**36.

[4] Vieux F, Darmon N, Touazi D, Soler LG. Greenhouse gas emissions of self-selected individual diets in France: changing the diet structure or consuming less? Ecol Econ 2012(75)91**–**101.

[5] Herring, G. (19 May, 2015). Meat industry has a cow over US dietary guidelines. *The Guardian*. Retrieved from [*http://www.theguardian.com/lifeandstyle/2015/may/19/us-dietary-guidelines-health-and-human-services-tom-vilsack*](http://www.theguardian.com/lifeandstyle/2015/may/19/us-dietary-guidelines-health-and-human-services-tom-vilsack)

[6] Hallström E, Carlsson-Kanyama A, Börjesson P. Environmental impact of dietary change: a systematic review. J Clean Prod 2014(91):1**–**11.

[7] Heller MC, Keoleian GA, Willett WCY. Toward a Life Cycle-Based, Diet-level Framework for Food Environmental Impact and Nutritional Quality Assessment: A Critical Review 2013(47).

[8] Joyce A, Hallett J, Hannelly T, Carey G. The impact of nutritional choices on global warming and policy implications: examining the link between dietary choices and greenhouse gas emissions. Energy and Emission Control Technologies 2014(2):33-43.

[9] Sanderson S, Tatt ID, Higgins JPT. Tools for assessing the quality and susceptibility to bias in observational studies in epidemiology: a systematic review and annotated bibliography. International Journal of Epidemiology 2007(36):666-676.
